# Editorial: Theoretical Issues on Sensory Perception—Approaches from Philosophy, Psychology, and Neuroscience

**DOI:** 10.3389/fpsyg.2017.01660

**Published:** 2017-09-22

**Authors:** Konstantinos Moutoussis

**Affiliations:** Department of History and Philosophy of Science, National and Kapodistrian University of Athens Athens, Greece

**Keywords:** theoretical issues, sensory perception, philosophy, psychology, neuroscience

One of the basic functions of the brain is connecting an animal to its environment. Evolution has developed several sensory systems for this purpose, each one using a different approach to give the agent a “picture” of the physical world. Inevitably, the way that we perceive our environment via these systems has always been a source of deep theoretical questions and problems concerning this very basic characteristic of our existence. It has been an interdisciplinary topic of research for many years: Philosophy is concerned with questions at the core of sensory perception, regarding the nature of perceptual events, as well as their phenomenal characteristics and epistemological value. Why does perception feel the way it does, and how successful is it in informing us about the world out there? Psychology has introduced several experimental methods in order to study the characteristics of sensory perception, as well as the relationship between the physical characteristics of the physical world and the psychological characteristic of the mental world. It uses results from behavioral experiments on sensory perception to infer knowledge about the organization and functioning of the underlying mechanisms of the mind. Finally, neuroscience is continuously struggling to understand these mechanisms by directly studying their neurobiological origins in the brain. The ultimate aim of cognitive neuroscience is to relate neuronal events taking place inside our skull to mental events and experiences happening in what we call the mind. Although we are far from reaching this endpoint, one might expect that the advances in the field of sensory neuroscience and our understanding of both the structure and functioning of perceptual systems, combined with our accumulated scientific knowledge regarding the behavioral characteristics of perception from the point of view of experimental and cognitive psychology, should put us today in a better place with respect to basic theoretical issues and fundamental philosophical questions regarding perceptual experiences.

The purpose of the current Special Topic is to bring together contributions from the fields of philosophy, psychology, and neuroscience, all of which address theoretical issues on the nature and functioning of sensory perception. Moutoussis addresses several philosophical issues regarding perception, from a neurobiological point of view. He argues that many of the problems raised in the philosophical literature emerge from using a language structured to describe events in the physical world to talk about evens in the mental world. Such a problem is the hypothetical separation between a percept and its content, which creates unnecessary complications regarding the nature of the “object” of perception. A distinction between physical and mental properties is made, and the way in which subjective perceptual experiences can provide objective knowledge is discussed. Different types of perceptual experiences are reviewed, with an emphasis on whether a percept *per se* can be characterized as being true or not. Shipp explores the neuronal circuitry supporting the popular theory of predictive coding in perception. Instead of hierarchical processing of the sensory input being the main computational architecture of the brain, predictive coding suggests a Bayesian, generative model using past experience to predict the sensory data. Errors between top-down predictions and bottom-up signals are calculated and ascend the hierarchy to optimize the former. The theory can explain several behavioral characteristics of perception but confronts a rich cortical microcircuitry that is yet to be fully documented. Knowledge regarding the latter in primate visual cortex, as well as technical advances using neural engineering in transgenic mice is evaluated to suggest a schematic template for the implementation of predictive coding in the brain. Purves et al. introduce a theoretical framework on how the visual system creates perceptual experience. The classical view has been that the former recovers features of the world to generate the latter. In the alternative view put forward here, vision assigns perceptual qualities empirically by associating frequently occurring stimulus patterns with useful responses, on the basis of survival success. Its function is not to make inferences regarding the physical properties of the stimulus, but to create a function that relates perceptual values to the frequency of recurring stimulus patterns. The challenge can be met by responses determined entirely by trial and error that eventually align perceptions with the frequency of occurrence of stimuli, on the basis of rewarded associations. MacKisack et al. review long-standing theoretical issues regarding visual imagery, in the light of findings from the field of contemporary neuroscience, and pose conceptual and methodological challenges to the scientific analysis from the perspective of the traditional theoretical positions. They concentrate on the act of imagining a physical object and take the reader through a detailed history of the philosophy and psychology of the subject. Emphasis is given on the critique of the mere existence of imagery and mental objects, as well as to the long standing debate regarding its iconic or conceptual nature. The latter iconophobic approach seems to be less popular today thanks, among others, to brain imaging studies revealing common neuronal mechanisms between imagery and perception. Finally, Chen and Spence review the unity assumption/effect in multisensory perception, that is, cases in which unisensory percepts from different modalities appear to arise from a common object or event in the real world. They critically assess the role of experimental instructions, redundant information, crossmodal correspondences, semantic congruency, and context in achieving the unity effect, and review experimental evidence from studies on spatial and temporal ventriloquism, the McGurk effect, and the Colavita visual dominance effect. The possible relationship between the nowadays popular Bayesian framework of brain function and the unity assumption is also discussed. They conclude that, despite several outstanding issues yet to be resolved, the latter clearly influences multisensory integration across a range of stimulus pairs from multiple sensory modalities.

In conclusion, the papers presented in this Research Topic shed new light to different, long-standing, theoretical issues on sensory perception. They nicely demonstrate the broadness of the field in terms of the ways by which it can be approached, thus suggesting the necessity of a multidisciplinary approach in understanding this very basic and important brain function.

## Author contributions

The author confirms being the sole contributor of this work and approved it for publication.

### Conflict of interest statement

The author declares that the research was conducted in the absence of any commercial or financial relationships that could be construed as a potential conflict of interest.

